# Plant-protein non-dairy creamers: A comprehensive review of structural modifications and processing innovations for improved performance

**DOI:** 10.1016/j.fochx.2026.104099

**Published:** 2026-06-13

**Authors:** Xuteng Wang, Bingyu Chen, Chao Yan, Huiwen Guan, Mengying Liu, Ning Li, Hongzhi Liu

**Affiliations:** a Key Laboratory of Geriatric Nutrition and Health (Beijing Technology and Business University), Ministry of Education, China; b GNC (Shanghai) Food Products Technology Co., Ltd., China

**Keywords:** Plant protein, Non-dairy creamer, Structure modulation, Functional properties

## Abstract

Non-dairy creamers are spray-dried oil-in-water emulsion powders that must provide rapid wetting, whitening, emulsion stability, oxidative protection, reconstitution and acceptable flavor. Replacing sodium caseinate with plant proteins is attractive for plant-based and sustainability-oriented formulations, but remains difficult because many plant proteins show poor solubility, slow interfacial adsorption, weak or overly rigid films, heat- or salt-induced aggregation and off-flavors. This review critically summarizes peer-reviewed studies on plant-protein-stabilized emulsions, lipid microencapsulation and spray-dried creamer-related powders, and, unlike general plant-protein or encapsulation reviews, focuses on how protein source, structural modification, oil loading, wall composition and processing jointly determine product performance within an emulsion-to-powder framework. Physical, enzymatic and chemical modifications can improve interfacial functionality, but excessive processing may impair stability, color, digestibility or sensory quality. Future work should use multivariate optimization and validate plant-protein creamers in realistic coffee or tea systems.

## 1 Introduction

**Fig. 1:**
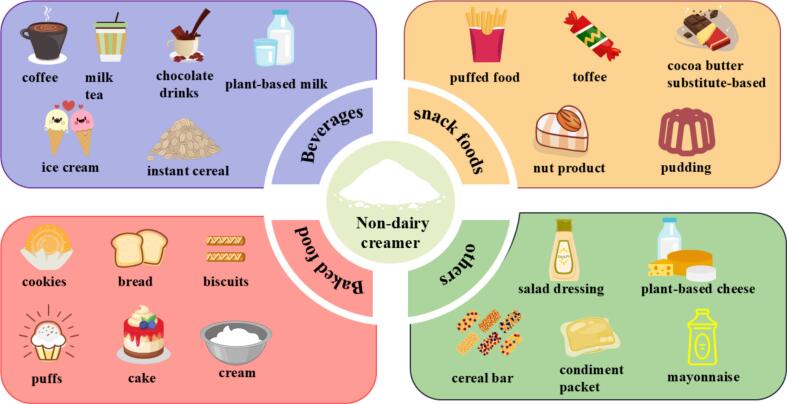
Non-dairy creamer and its multidimensional applications.

Non-dairy creamers are spray-dried oil-in-water emulsion powders widely used in coffee, tea drinks, milk tea, instant beverages and bakery products (Fig. 1). Their quality is not determined only by solubility, but by the combined performance of wetting, whitening, emulsion stability, oxidative resistance, powder flowability, reconstitution behavior and sensory acceptability. In conventional formulations, sodium caseinate is widely used as a functional protein and wall material because it adsorbs efficiently at the oil–water interface, helps form protective interfacial films and contributes to the stability and whitening effect of creamer emulsions (Wang et al., 2024; Wang & Liu, 2014). However, its dairy origin, potential allergenicity and reliance on animal-derived resources have become inconsistent with the growing demand for plant-based and sustainability-oriented formulations. Previous work on non-dairy creamer and oil microencapsulation has shown that protein source, wall material, oil loading, total solids and drying conditions all affect encapsulation efficiency, oxidation stability and final powder quality, suggesting that creamer performance should be understood as an integrated emulsion-to-powder process rather than as the result of a single ingredient (Liu et al., 2023; Mohammed et al., 2020; Rosida et al., 2016).

**Fig. 2:**
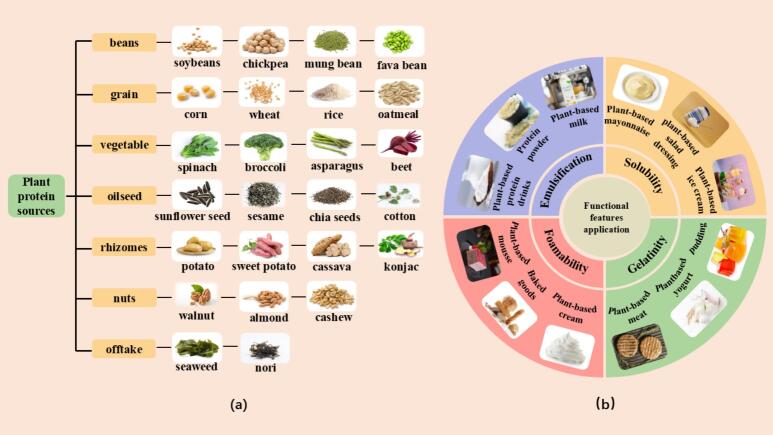
Representative plant-protein sources (a) and their corresponding food applications (b).

Replacing sodium caseinate with plant proteins is attractive but remains technically unresolved. Plant proteins from legumes, cereals, oilseeds, nuts and seaweeds offer advantages such as broad availability, lactose-free and cholesterol-free characteristics, and potential nutritional or bioactive value (Fig. 2). Recent studies on plant-derived proteins, including seaweed protein-derived bioactive peptides and protein-enriched plant-based or hybrid frozen desserts, further show the growing interest in using non-dairy proteins to improve nutritional and functional quality (Basri et al., 2025; Phumsombat et al., 2024; Singh et al., 2025; Xiao et al., 2023).

Nevertheless, the functionality required in non-dairy creamers is more demanding than simple protein enrichment. Many native plant proteins have low solubility near neutral or mildly acidic pH, compact globular structures, strong protein–protein aggregation, slow interfacial adsorption, weak or brittle interfacial films, and characteristic off-flavors. These limitations can lead to droplet flocculation, coalescence, feathering in hot coffee or tea, increased surface oil after spray drying, poor wettability and reduced consumer acceptance. Therefore, the central challenge is not whether plant proteins can act as emulsifiers, but how their structure can be tuned to reproduce the interfacial, encapsulation and reconstitution functions traditionally provided by sodium caseinate (Islam et al., 2023).

Structural modification provides a practical route to narrow this functionality gap, because physical, enzymatic and chemical treatments can regulate the structure–function relationship of plant proteins and thereby improve their emulsifying and encapsulating performance. Physical treatments such as heating, ultrasound, high-pressure processing and high-pressure homogenization can alter aggregation state, expose buried hydrophobic groups and improve molecular flexibility; however, their effects depend strongly on treatment intensity, protein source and environmental conditions (D'Alessio et al., 2025; Li, Chen, et al., 2025). Enzymatic hydrolysis can enhance solubility and diffusion to the interface, although excessive hydrolysis may generate bitter peptides or weaken interfacial film cohesion, indicating that the degree of hydrolysis should be carefully controlled (Vogelsang-O'Dwyer et al., 2022; Wang, Chen, et al., 2025). Chemical modifications such as glycation, phosphorylation and acylation can modify hydration, electrostatic repulsion and protein–oil affinity, but they may also introduce trade-offs related to browning, digestibility, sensory quality and regulatory acceptability (Shen & Li, 2021; Zhao et al., 2022). Thus, the performance of modified plant proteins in creamers should be interpreted through a mechanism-based framework linking molecular conformation, adsorption kinetics, interfacial rheology, emulsion stability, spray-drying behavior and powder reconstitution, rather than through isolated indicators such as solubility or emulsifying activity alone. This point is especially important because studies on plant-protein emulsions often differ in protein source, degree of modification, oil loading, wall composition, homogenization intensity, total solids and drying conditions, making direct comparison difficult (Raquel Reis et al., 2023; Zhang et al., 2023).

Building on these considerations, this review examines plant-protein non-dairy creamers as an integrated emulsion–powder system rather than as a simple replacement of one protein source by another. First, the structural characteristics and functional limitations of native plant proteins are discussed in relation to their solubility, molecular flexibility, interfacial adsorption and film-forming ability. Then, physical, enzymatic, chemical and fermentation-related modification strategies are compared in terms of how they regulate protein conformation, aggregation behavior and techno-functional performance. Particular attention is given to the connection between modified protein structure and key creamer-related properties, including emulsion stability, encapsulation efficiency, surface oil, oxidative stability, powder dispersibility and reconstitution behavior. In addition, formulation and processing factors such as oil phase selection, wall material composition, protein concentration, pH, ionic strength, homogenization and spray drying are considered as interacting variables rather than isolated parameters. By linking protein modification mechanisms with emulsion formation, powder production and end-use performance, this review aims to provide a clearer framework for the rational design of plant-protein non-dairy creamers and to identify practical challenges that should be addressed in future studies.

## 2 Effects of modifications to plant proteins

Plant proteins are often limited by poor aqueous solubility, slow interfacial adsorption, weak film-forming capacity, aggregation under heat or ionic stress, and undesirable sensory attributes such as bitterness or beany notes. These limitations restrict their direct use as functional proteins and wall materials in oil-in-water emulsions and spray-dried creamer powders (Nasrabadi et al., 2021). Structural modification is therefore widely used to improve solubility, molecular flexibility, surface activity, interfacial film formation and encapsulation performance. However, the reported effects of different modification methods cannot be compared only by the final value of encapsulation efficiency or emulsion stability, because studies often differ in protein source, core oil, wall composition, oil loading, total solids, homogenization intensity and spray-drying conditions (Zhang et al., 2023). A more meaningful comparison should connect the modification method with both feed-emulsion properties and powder-level performance (Table 1). For example, Nesterenko et al. (2014) compared native and modified soybean and sunflower proteins as wall materials for spray-dried α-tocopherol microencapsulation under the same experimental system. Among hydrolysis, acylation, cationization and cross-linking, acylation gave the highest core retention, reaching 94.8–99.5%, which was attributed to stronger affinity between the hydrophobic core and the modified protein wall. This suggests that chemical modification can be effective when protein–core affinity is the main limiting factor. However, such results should not be directly compared with studies using different oils, carrier matrices or drying conditions (Gouveia Gomes & Kurozawa, 2020).

**Table 1:** Category-specific comparison of plant-protein modification strategies: structural changes, functional outcomes, and relevance to non-dairy creamer systems.

Modification category	Specific method	Protein source	Main structural change	Functional improvement	Nutritional / sensory / safety implication	Relevance to plant-protein non-dairy creamers	References
Thermal treatment	Wet heating; dry heating	Soy, pea and walnut, proteins	Partial unfolding, exposure of hydrophobic groups, aggregate rearrangement; excessive heating may cause irreversible aggregation.	May improve interfacial adsorption, thermal stability and film formation when denaturation is mild and controlled.	Possible cooked flavor, browning, digestibility changes or loss of heat-sensitive nutrients under excessive treatment.	Useful as a pretreatment before emulsification, but aggregation before spray drying should be avoided.	(Shen et al., 2022; Ye et al., 2025; Zhao, Ren, et al., 2024)
Non-thermal physical treatment	Ultrasound	Pea, soy, peanut and walnut proteins	Aggregate disruption, reduced psize, exposure of buried hydrophobic groups and modification of surface charge.	Improved dispersion, solubility, interfacial adsorption and emulsifying properties in many systems.	Excessive sonication may promote re-aggregation, oxidation or loss of interfacial film strength.	Helpful for improving plant-protein dispersion and oil-water interfacial adsorption before homogenization.	(Du et al., 2025; Shi et al., 2023; Yan et al., 2024)
	High-pressure processing (HPP)	Pea, soy, rice and walnut proteins	Disruption of non-covalent interactions, unfolding, dissociation of aggregates; excessive intensity may induce aggregation.	Can reduce por droplet size, increase interfacial area and promote protein adsorption.	High energy input and temperature rise may cause denaturation, lipid oxidation or weakened interfacial layers.	Directly relevant to creamer emulsion formation and homogenization, but optimization must consider oil loading and wall composition.	(Li et al., 2023; Shi et al., 2023)
	Microjet / microfluidization	Walnut, soy, pea or other plant proteins	Strong shear, collision and cavitation reduce aggregate size and alter tertiary/quaternary structures.	Improves aqueous dispersion and can enhance emulsifying activity when over-processing is avoided.	Possible over-shearing, protein aggregation or loss of functional balance at high intensity.	Potential pretreatment for plant-protein ingredients before preparation of feed emulsions.	(Kerezsi et al., 2024)
Enzymatic hydrolysis	Alcalase, Flavourzyme, trypsin, papain or mixed proteases	Pea, lentil, soy, rice, chickpea and cereal/legume proteins	Peptide-bond cleavage, lower molecular weight, more ionizable groups and exposure of hydrophobic segments.	Moderate hydrolysis can improve solubility, diffusion to the interface and interfacial mobility.	May improve digestibility and release bioactive peptides; excessive hydrolysis may generate bitter peptides and weaken film cohesion.	Useful for improving interfacial mobility, but hydrolysis degree must be controlled to maintain oil retention and powder stability.	(Ye et al., 2025)
Chemical modification	Maillard-type glycation	Soy, pea, rice, walnut and other plant proteins	Covalent protein-carbohydrate conjugates, improved hydration, steric repulsion and interfacial layer thickness.	May improve solubility, emulsifying stability, thermal resistance and encapsulation potential.	Functional gains should be balanced against browning, flavor change, digestibility loss and advanced glycation concerns.	Potentially useful for stabilizing creamers under heat/salt stress, but reaction extent must be controlled.	(El Hosry et al., 2025)
	Phosphorylation	Perilla, pea, peanut, soy and other plant proteins	Increased negative charge, stronger hydration, altered conformation and improved electrostatic repulsion.	Improves solubility, dispersion and emulsion stability, especially where electrostatic stabilization is important.	Reagent use, residual phosphates, regulatory acceptance and clean-label perception should be considered.	May improve resistance to aggregation in beverage matrices, but compatibility with salts and pH should be tested.	(Tang et al., 2024)
	Acylation	Soy, sunflower, pea and other plant proteins	Altered amphiphilicity, increased protein-oil affinity, modified charge and molecular packing.	Can improve oil affinity, oil retention and encapsulation when lipid-core affinity limits performance.	Chemical reagent acceptability, clean-label limitations and excessive hydrophobicity should be considered.	Relevant to oil-rich spray-dried creamer powders, especially for reducing surface oil.	(Tang et al., 2024)
Fermentation modification	Lactic acid bacteria fermentation	Pea, soy, quinoa, millet, cereal and legume proteins	Microbial proteolysis, acidification, enzyme production, reduced antinutritional factors and altered surface properties.	May improve solubility, digestibility, flavor quality and sometimes emulsifying properties.	May reduce beany flavor or antinutritional factors; results are strongly strain- and substrate-dependent.	Promising for ingredient pretreatment, but creamer-specific validation in hot coffee/tea systems is still needed.	(Cirunay et al., 2026)
	Fungal fermentation / mixed-culture fermentation	Cereal, legume, oilseed or by-product proteins	Proteolysis, cell-wall disruption, metabolite production and flavor-active compound formation.	Can improve nutritional availability, digestibility and sensory quality; functional effects vary by substrate.	Excessive fermentation may cause off-flavor, acidification or inconsistent functionality.	May help reduce off-flavor and improve nutritional profile of plant-protein ingredients before creamer formulation.	
Combined modification	pH-shifting + ultrasound; ultrasound + HPH; enzymatic hydrolysis + glycation	Pea, soy, peanut, mung bean, walnut and other plant proteins	Synergistic unfolding, aggregate disruption, exposure of active groups, peptide formation or conjugation.	May balance solubility, interfacial adsorption, film strength and steric/electrostatic stabilization.	Higher cost, over-processing risk and lower reproducibility should be considered.	Promising for plant-protein creamer systems, but should be validated across emulsification, spray drying and reconstitution.	(Tang et al., 2024)

The importance of formulation context is also evident in enzymatic hydrolysis. Bajaj et al. (2017) used pea protein hydrolysates for flaxseed oil microencapsulation and found that the best 60-min hydrolysate achieved a microencapsulation efficiency of 56.2% at 20% oil loading, whereas efficiency decreased at higher oil loading. When native pea protein and maltodextrin were incorporated, microencapsulation efficiency increased to above 90%, indicating that wall composition and oil loading may be as important as hydrolysis itself. Similarly, rice protein hydrolysates prepared with different proteases showed distinct functions in linseed oil emulsions: Flavourzyme hydrolysates favored smaller and more stable droplets, whereas Alcalase hydrolysates contributed more to oxidative protection. These findings indicate that enzymatic hydrolysis may improve solubility and interfacial diffusion, but its practical benefit depends on the degree of hydrolysis, peptide composition and target endpoint (Gouveia Gomes & Kurozawa, 2021; Vogelsang-O'Dwyer et al., 2022).

Recent studies on plant-based spray-dried powders further support the need for normalized comparison. Plant-based high-oil powders prepared with mung bean protein and maltodextrin showed that both oil loading and initial emulsion droplet size affected surface oil loading, powder clustering, bulk density and reconstitution behavior. Powders prepared from smaller oil droplets and 20% oil loading showed better physicochemical properties than those produced from larger droplets or higher oil loading (Adhikari et al., 2025). In another study, hemp, canola and flax seed proteins combined with maltodextrin produced fully plant-based spray-dried powders, but encapsulation efficiency, morphology, psize, hygroscopicity and storage stability differed among protein sources (Ben-Othman et al., 2024). Overall, different modification strategies exert distinct and sometimes competing effects on plant protein structure and functionality. Therefore, modification methods should be evaluated in relation to both feed-emulsion properties and powder-level endpoints. For plant-protein non-dairy creamers, the most relevant comparison should extend beyond solubility and emulsifying activity to include encapsulation efficiency, surface oil, oxidative stability, flowability, wettability, dispersibility and reconstitution stability in coffee or tea matrices (Heiden-Hecht et al., 2021).

### 2.1 The effect of different modification methods on the structure of plant proteins

**Fig. 3:**
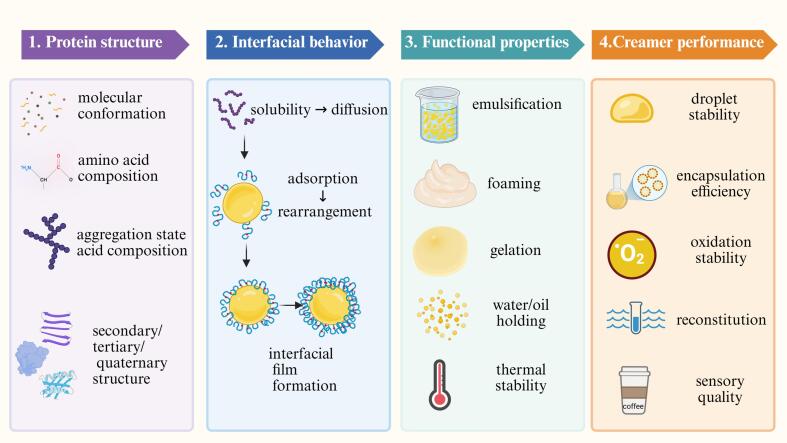
Mechanistic links between plant-protein structure, interfacial behavior and creamer-related functionality.

Protein structure determines not only the solubility and dispersion of plant proteins in the aqueous phase, but also their diffusion, adsorption and rearrangement at the oil–water interface. Therefore, changes in α-helix, β-sheet, β-turn, random-coil content or fluorescence spectra should be interpreted as evidence of conformational rearrangement rather than as direct proof of improved creamer performance. For plant-protein non-dairy creamers, structural modification should be understood through a mechanism-based pathway: molecular conformation affects solubility and diffusion; diffusion and surface activity determine adsorption kinetics; interfacial rearrangement determines the viscoelasticity and strength of the adsorbed film; and the interfacial film controls droplet stability during emulsification, homogenization and spray drying. These mechanistic links are summarized in Fig. 3. This is important because plant-protein emulsifying performance depends not only on bulk structure but also on adsorption behavior, interfacial film formation and resistance to environmental stresses (Kontogiorgos & Prakash, 2023; Zhang et al., 2023).

Many native plant proteins are compact globular proteins or multimeric aggregates with strong protein–protein interactions, which may limit molecular flexibility and slow adsorption at newly formed oil–water interfaces. Physical, enzymatic and chemical modifications can dissociate aggregates, expose buried hydrophobic groups, alter surface charge and improve molecular flexibility. However, these effects are not always beneficial. Moderate unfolding may promote interfacial adsorption and film formation, whereas excessive unfolding, over-hydrolysis or aggregation may reduce solubility, weaken interfacial viscoelasticity or form brittle films. For example, high-pressure homogenization can alter pea protein structure and interfacial behavior, but stronger treatment does not necessarily improve emulsion stability because the adsorbed layer may become less elastic or less homogeneous (D'Alessio et al., 2025). This structure–interface relationship is directly linked to powder performance in spray-dried non-dairy creamers. If the interfacial layer around oil droplets is incomplete or mechanically weak, droplets may flocculate, coalesce or migrate toward the psurface during drying, increasing surface oil and accelerating oxidation. In contrast, a stable protein or protein–polysaccharide interfacial layer can improve droplet protection before drying and work with carbohydrate wall materials to support powder formation. Therefore, the evaluation of modified plant proteins should combine bulk structural indicators with interfacial and powder-level endpoints, including dynamic interfacial tension, interfacial rheology, droplet size, encapsulation efficiency, surface oil, oxidative stability, wettability, dispersibility and reconstitution behavior (Adhikari et al., 2025; Ben-Othman et al., 2024).

### 2.2 Effects of different modification methods on the functional properties of plant proteins

#### 2.2.1 Solubility

Solubility is a key indicator of protein processability because it reflects the balance between protein–water interactions and protein–protein associations. Strong hydration and sufficient electrostatic repulsion generally favor dispersion in aqueous systems. Adequate solubility is often a prerequisite for evaluating functional properties, including foaming, emulsifying, and gelling behaviors (Ma et al., 2022). However, many plant proteins exhibit limited solubility under near-neutral conditions. Rice protein is a typical example: rice endosperm storage proteins are dominated by glutelin, which is mainly alkali/acid-soluble, whereas prolamin is alcohol-soluble and usually represents a minor fraction. Therefore, native rice protein ingredients often show poor dispersibility and lower solubility at pH ∼7, depending on the raw material and processing history. Improving solubility can expand the use of plant proteins in non-dairy creamers (Yue et al., 2024). High-pressure treatments can modify solubility by perturbing non-covalent interactions, dissociating protein assemblies (Kang et al., 2022). Notably, the direction and magnitude of the solubility change are highly condition-dependent. Excessive unfolding can expose hydrophobic patches and promote aggregation, which may offset solubility gains. During Maillard-type glycation, soy protein isolate can covalently bind carbohydrate moieties rich in hydroxyl groups. This typically increases hydrophilicity and steric stabilization, loosens compact structures, and improves solubility (Chen et al., 2024). Li et al. (2023) reported that high-pressure microjet treatment markedly increased walnut protein solubility. This effect is commonly attributed to reduced aggregate size and increased surface charge generated by intense shear and rapid pressure release, both of which favor aqueous dispersion. Thus, solubility should be treated as a prerequisite for dispersion and interfacial diffusion, while practical stability should be assessed through interfacial film formation and stress resistance. Over-unfolding or over-hydrolysis may increase apparent solubility but weaken the formation of cohesive, viscoelastic interfacial films required to resist coalescence during heating or in high-ionic-strength beverage matrices. Therefore, solubility should be interpreted together with interfacial activity and stability metrics measured under application-relevant conditions.

#### 2.2.2 Emulsifying property

The emulsifying property of plant proteins refers to their ability to stabilize oil-water mixtures to form emulsions, which is critical to the texture and stability of plant protein non-dairy creamers. Proteins stabilize oil-in-water emulsions through a multistep interfacial process that includes diffusion from the bulk, adsorption/penetration into the interface, and subsequent conformational rearrangements that build a viscoelastic interfacial film. Plant proteins often form weaker films than dairy proteins because their globular, compact and frequently aggregated colloidal states limit unfolding and interfacial network formation; therefore, modification strategies that increase molecular flexibility while preserving interfacial elasticity are particularly relevant. Different modification methods can improve the emulsifying property of plant proteins by changing their structure and surface properties (Sarathy et al., 2025).

Maillard-type glycation, or protein–carbohydrate conjugation, can improve plant-protein emulsifying performance by increasing hydrophilicity, steric repulsion and interfacial layer thickness, thereby enhancing solubility, droplet stabilization and resistance to pH, salt or heat stresses. These advantages make glycation useful for plant-protein-stabilized creamer emulsions, especially when native proteins form weak or unstable interfacial films. However, its benefits depend strongly on reaction control. Excessive Maillard reaction may cause browning and flavor changes, promote the formation of advanced glycation end-products, reduce reactive lysine availability and impair protein digestibility. Therefore, glycated plant proteins should be evaluated not only by emulsifying activity or droplet size, but also by grafting degree, browning index, AGE markers, in vitro digestibility and sensory performance in coffee or tea systems (El Hosry et al., 2025; Kutzli et al., 2021). In addition, a combined pH-shifting–ultrasonication–heating strategy substantially increased pea protein solubility and enhanced the stability of sunflower oil-in-water emulsions, consistent with aggregate disruption and exposure of interfacially active groups (Zhi et al., 2022). Overall, diverse modification approaches provide feasible routes for improving plant proteins as emulsion stabilizers. However, improved emulsifying activity does not necessarily translate into long-term stability. Storage stability is governed by interfacial viscoelasticity, continuous-phase rheology, and resistance to environmental stresses. Therefore, emulsions should be evaluated using standardized stress tests (e.g., heating, freeze–thaw cycling, and ionic strength/pH challenges) that reflect realistic processing and application conditions.

#### 2.2.3 Foaming and foaming stability

Proteins exhibit amphiphilic properties and can act as surfactants in aerated systems. During whipping, protein molecules diffuse through the aqueous phase and adsorb at the air–water interface, with hydrophilic regions remaining in the aqueous phase while hydrophobic residues preferentially orient toward the air phase. This adsorption reduces surface tension, facilitating air incorporation and bubble formation. Following adsorption, proteins partially unfold and form lateral non-covalent protein-protein interactions, generating a thin, mechanically resilient interfacial layer that supports foam formation and stability (Chatzigiannakis et al., 2025). Surface hydrophobicity and electrostatic interactions further regulate adsorption kinetics and interfacial layer structure. Enhanced surface hydrophobicity typically increases interfacial adsorption driving forces, accelerating surface coverage and thereby promoting rapid foam formation. Concurrently, moderate charge shielding (e.g., via pH or ionic strength adjustment) reduces electrostatic repulsion between adsorbates, promoting close packing and interfacial network formation. However, excessive charge reduction may induce flocculation and compromise foam stability. These determinants can be optimized through process control to enhance foaming performance (Amagliani et al., 2021). For example, Li et al. (2023) reported that ultra-fine grinding reduced walnut protein psize and increased specific surface area, which promoted adsorption at the air–water interface and the formation of stable interfacial films, ultimately improving foaming capacity and foam stability. Foaming performance is often measured using protein solutions under standardized whipping conditions. However, practical non-dairy creamer formulations also contain oil droplets, sugars, and low-molecular-weight surfactants that may compete for the interface or disrupt protein films. Therefore, foaming tests should be selected to match the target application (e.g., whipped toppings versus coffee creamers), and potential trade-offs between foaming and emulsifying functionality should be evaluated.

#### 2.2.4 Thermal stability

Thermal stability dictates whether plant proteins retain solubility and interfacial functionality during high-temperature operations, including hot emulsification and spray drying. Heat-induced unfolding and aggregation can compromise emulsifying performance and impair powder rehydration, whereas interventions that suppress irreversible aggregation tend to improve redispersibility after drying (Devaki & Ghosh, 2024; Nasrabadi et al., 2025). For instance, a combined media-milling/pH-shifting/heat pretreatment markedly increased the post-drying solubility of pea protein isolate and yielded powders that redispersed while maintaining a fine particle-size distribution (Li, Yan, et al., 2025). Maillard-type conjugation is another route to enhance heat tolerance at interfaces. Ashaolu and Zhao (2020) micronized okara dietary fibre and conjugated it with soy protein isolate via a dry-heating Maillard reaction (60 °C), producing ODF–SPI conjugates with good thermal stability and strong Pickering emulsion-stabilizing potential. Similarly, Sun et al. (2022) reported that glucose-mediated glycation of pea protein isolate enhanced emulsification efficiency and improved both thermal and oxidative stability of Pickering high-internal-phase emulsions in a glycation-extent-dependent manner. Despite these benefits, thermal processing and Maillard reactions may also promote aggregation and non-enzymatic browning, with concomitant color development and flavor changes during spray drying. Therefore, thermal-stability optimization should be balanced against sensory constraints and verified under process-relevant heating–drying–rehydration cycles that mimic spray drying and downstream use.

#### 2.2.5 Water holding capacity and oil holding capacity

Water holding capacity is identified as the amount of water retained per gram of protein, reflecting its ability to hold water against gravity, which affects food texture, juiciness, and shelf life. It is also termed water absorption, binding, or hydration capacity. Oil holding capacity, also called oil absorption or binding capacity, is the amount of oil retained per gram of protein through its hydrophobic side chains. The water absorption capacity of proteins is influenced by the distribution of polar amino acids at protein–water interaction sites, whereas the oil absorption capacity is determined by the location of nonpolar amino acids at protein–oil interaction sites. These two properties can effectively reduce moisture loss, thereby improving food texture and enhancing taste (Olatunde et al., 2026; Tarahi et al., 2024). Dong et al. (2024) found that radiofrequency treatment can improve the water absorption and oil absorption properties of indica-japonica hybrid rice protein. Zhu et al. (2017) found that high-pressure treatment at 100 and 200 MPa significantly increased the solubility and oil absorption of rice bran protein, while water absorption and foaming capacity further increased, reaching a maximum at 500 MPa. Although water- and oil-holding capacities are widely reported, their measured values depend strongly on assay conditions (e.g., centrifugation force, psize, and ionic strength). Moreover, for non-dairy creamers these bulk binding indices should be connected to application-relevant outcomes such as powder flowability, reconstitution kinetics, and resistance to oiling-off, rather than being interpreted as stand-alone indicators of product quality.

#### 2.2.6 Sensory characteristics

The sensory quality of plant-protein non-dairy creamers should be evaluated as a product-level attribute rather than inferred only from protein hydrolysis or bioactive peptide formation. Plant proteins commonly carry beany, grassy, earthy, bitter or astringent notes, which can originate from both raw materials and processing. In pea and soy proteins, off-flavors are often associated with lipid oxidation and volatile compounds such as aldehydes, alcohols, ketones, furans and pyrazines, including hexanal, heptanal, 2-pentylfuran and unsaturated aldehydes. These compounds may be retained or released differently depending on protein source, extraction conditions, lipid residues, heat treatment and fermentation. Therefore, sensory improvement should be supported by chemical analysis of off-flavor compounds, such as GC–MS, GC–IMS or GC–olfactometry, rather than by descriptive claims alone (Serap et al., 2024; Sharma et al., 2025). Enzymatic hydrolysis can improve solubility and interfacial activity, but it may also increase bitterness when hydrophobic peptides accumulate. The bitterness of protein hydrolysates is related not only to peptide concentration, but also to peptide sequence, molecular weight, hydrophobic amino-acid content and sensory threshold. Recent work on plant-protein hydrolysates showed that increasing the degree of hydrolysis can increase bitter-peptide release and reduce bitterness thresholds, indicating that hydrolysis should be optimized for both functionality and sensory acceptability. For plant-protein creamers, sensory evaluation should therefore include trained descriptive analysis or rapid profiling methods such as RATA/CATA, together with consumer acceptance or hedonic testing. Key attributes should include beany/grassy aroma, bitterness, astringency, aftertaste, mouthfeel, whitening, feathering and overall liking (Liu et al., 2022; Vaikma et al., 2022). Matrix-specific testing is particularly important because sensory defects may change after powder reconstitution and addition to hot coffee or tea. Heat, salts, coffee acids, polyphenols and ionic strength can alter protein aggregation, aroma release, mouthfeel and emulsion stability. Thus, a plant protein that shows acceptable flavor in dilute solution may still produce bitterness, beany notes, feathering or poor mouthfeel in a real creamer matrix. Future studies should validate modified plant proteins in reconstituted powders and hot coffee/tea systems, and should combine sensory scores with instrumental endpoints such as volatile profiles, droplet stability, surface oil, whitening capacity and reconstitution behavior (Appiani et al., 2023).

## 3 Preparation of plant protein non-dairy creamer emulsion

### 3.1 Preparation of raw materials

#### 3.1.1 Wall materials

The primary role of wall materials is to form a protective barrier between the core and the external environment, thereby reducing premature interactions, limiting volatilization, and enabling controlled release under defined conditions. Accordingly, wall-material selection governs not only the stability of the feed emulsion prior to drying, but also downstream encapsulation efficiency, oxidative protection, and the functional attributes of the resulting microcapsules. An ideal wall material needs to exhibit high water solubility, low solution viscosity at practical solids contents, and good film-forming ability; sufficient emulsifying capacity is also required to generate a stable emulsion before spray drying. Although proteins are typically minor components in non-dairy creamers, they strongly affect viscosity, emulsion stability, and reconstitution behavior. Proteins can serve as effective spray-drying wall materials because they adsorb at oil–water interfaces and bind flavor and lipid compounds (Mohammed et al., 2020). Sodium caseinate is widely used in conventional spray-dried systems. However, it may aggregate or precipitate under acidic conditions, particularly as pH approaches its isoelectric region, which restricts its use in acidified beverages. Recent work indicates that plant proteins can also serve as coating agents (Table 2), offering a pathway toward dairy-free encapsulation systems.

**Table 2:** Examples of different plant proteins used as wall materials.

Plant protein wall material	core material	Microcapsule encapsulation results	Reference
Pea protein isolate	Sunflower oil	Sunflower oil encapsulated in separated pea protein has good oxidative stability and helps improve emulsion stability.	(Le Priol et al., 2019)
Soy protein isolate	Paprika oleoresin	The retention rate of carotenoids in Paprika oleoresin has increased, and storage stability has been improved.	(Paola Rascon et al., 2011)
RuBisCo protein isolate	Soybean oil	Good emulsifying properties, helps form and stabilize oil-in-water emulsions.	(Tan et al., 2022)
Barley protein	Fish oil	It has a high ability to prevent oil oxidation, high oil encapsulation efficiency, loading efficiency, and low moisture content. It can form microcapsules with a dense and smooth surface.	(Wang et al., 2011)
Synergistic effects of pea protein and rice bran protein	Palm oil	Enhances the function of individual proteins to form stable emulsions that exhibit strong anti-flocculation properties at low temperatures.	(Tong et al., 2025)

Moving beyond single-component walls, plant proteins are often combined with polysaccharides to reinforce protein-stabilized interfaces. This approach is especially useful near the protein isoelectric point, or under higher ionic strength, where electrostatic stabilization weakens and droplets aggregate more readily (Islam et al., 2023). Depending on formulation and processing, these systems can form soluble electrostatic complexes, coacervates, Maillard-type conjugates, or multilayer interfacial architectures. In many cases, the polysaccharide moiety thickens and hydrates the interfacial layer. This creates steric and electrosteric repulsion. As a result, flocculation and coalescence are suppressed under stresses including acidification, salting, heating, and freeze–thaw. Nevertheless, excessive association may increase viscosity and trigger bridging-type flocculation, undermining emulsion stability and processability. Therefore, the biopolymer ratio, mixing order, and ionic conditions need to be tuned to the intended stabilization mechanism (Nooshkam et al., 2023). Wall systems based on mixed biopolymers often outperform single materials, and synergistic designs have broad potential for protecting sensitive cores in foods and related products (Gharsallaoui et al., 2007). However, many studies emphasize encapsulation efficiency as the primary endpoint, whereas for non-dairy creamers the practical performance is governed by additional attributes such as surface oil loading, oxidative stability during storage, powder flowability, and rapid reconstitution without oiling-off. To strengthen the evidence base, future work should report these application-relevant metrics and test microcapsules in realistic beverage systems where pH, salts, and polyphenols can destabilize protein-stabilized interfaces.

#### 3.1.2 Core material

Plant oils are not only lipid carriers in non-dairy creamers, but also key determinants of whitening, creaminess, mouthfeel, oxidative stability and flavor quality. Their performance depends on fatty-acid composition as well as refining history and minor lipid components. Oils rich in unsaturated fatty acids may provide a more favorable nutritional profile, but they are generally more susceptible to oxidation, which can generate off-flavors and reduce storage stability. Therefore, oil selection for plant-protein creamers should consider both nutritional value and oxidative behavior rather than fatty-acid composition alone (Aslam & Schroen, 2023; Ghelichi et al., 2023). Refining and degumming can strongly affect the behavior of oils in emulsion systems. Degumming removes phospholipids and other mucilaginous materials from crude oils, which may improve oil clarity, reduce processing instability and decrease impurities that affect flavor and shelf life. However, phospholipids are amphiphilic molecules and may also act as natural interfacial components. Their removal or transformation can therefore alter oil–water interfacial composition, droplet formation and the interaction between oil droplets, plant proteins and low-molecular-weight emulsifiers. Feng et al. (2026) showed that different degumming methods produced distinct changes in the structural profiles and physicochemical properties of Idesia polycarpa phospholipids; enzymatic degumming achieved high degumming efficiency and uniquely generated lysophosphatidic acid, indicating that the degumming route can reshape phospholipid composition rather than simply remove impurities (Gharby, 2022). Lipid minor components, including residual phospholipids, free fatty acids, monoacylglycerols, diacylglycerols, tocopherols, phytosterols, pigments and trace metals, may further influence creamer stability. Some components, such as tocopherols and certain phospholipids, may contribute antioxidant or interfacial effects, whereas free fatty acids, pigments or metal traces may accelerate oxidation or promote undesirable flavor development. These effects are especially relevant in spray-dried creamers because lipid oxidation and surface oil are closely linked to interfacial protection, powder matrix formation and storage conditions. Thus, future studies should report the refining/degumming status of oils, phospholipid residues, peroxide value, anisidine value or other oxidative indices, and should evaluate reconstituted creamers in hot coffee or tea matrices where salts, acids and polyphenols may destabilize protein-stabilized oil droplets (Feng et al., 2026; Samdani et al., 2018).

#### 3.1.3 Emulsifiers

**Fig. 4:**
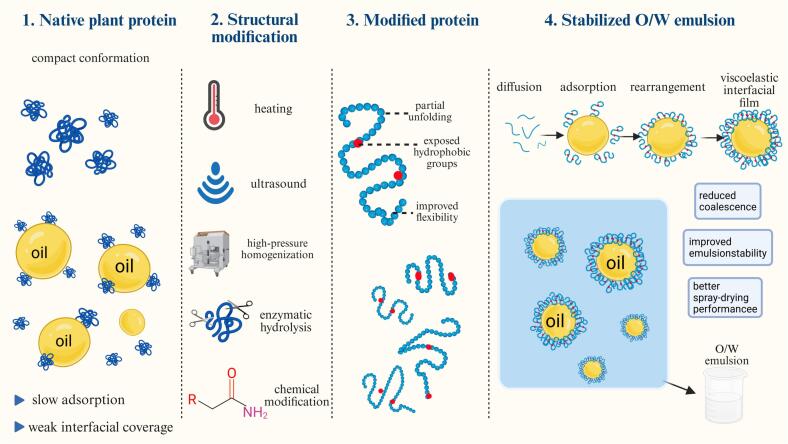
Stabilization mechanism of plant-protein-stabilized oil-in-water emulsions before spray drying.

Recent years have seen substantial progress in the research and application of plant proteins as food emulsifiers. Owing to their amphiphilic character, proteins can adsorb at oil–water interfaces, reduce interfacial tension, and form protective interfacial films that inhibit droplet coalescence (Nasrabadi et al., 2025). However, emulsifying performance varies markedly with protein source, structure, molecular weight distribution, and adsorption behavior. Compared with dairy proteins, many native plant proteins exhibit less favorable interfacial kinetics and weaker interfacial films, but these limitations can be mitigated through targeted modification and formulation strategies (Kim et al., 2020). Although proteins are typically minor ingredients in non-dairy creamers, they can strongly influence viscosity, emulsion stability, and reconstitution behavior (Zhang et al., 2023). Therefore, protein emulsification remains a key functionality in creamer design. As natural emulsifiers, proteins generally require partial conformational rearrangement upon adsorption to expose hydrophobic residues toward the oil phase while maintaining hydrophilic interactions with the aqueous phase, thereby stabilizing oil-in-water (O/W) emulsions (Zhang et al., 2022). Fig. 4 shows the stabilizing effect of proteins on oil droplets in oil-in-water emulsions. In a model non-dairy creamer system, Rosida et al. (2016) evaluated protein concentrates from leucaena, green bean, and red bean in combination with sodium caseinate (1.5–2.5%). A formulation containing 2.0% sodium caseinate and 1% green bean protein concentrate yielded the most preferred product and favorable physicochemical indices, supporting partial replacement of milk-derived protein in creamer applications. Overall, plant proteins can serve as emulsifiers, but their performance is highly context-dependent. Emulsifier selection should be guided by mechanistic understanding of interfacial adsorption and by validation in the final formulation, rather than by emulsifying-activity indices alone.

#### 3.1.4 Other ingredients

In plant-protein non-dairy creamers, stabilizers, low-molecular-weight emulsifiers, phosphate salts and plant proteins should not be considered as independent additives, because they jointly determine interfacial composition, droplet interactions and reconstitution behavior. Plant proteins can adsorb at the oil–water interface and form viscoelastic films, whereas low-molecular-weight emulsifiers, such as mono- and diglycerides or sucrose esters, can rapidly reduce interfacial tension and facilitate droplet breakup. However, excessive small-molecule emulsifiers may compete with or partially displace proteins from the interface, weakening protein-based interfacial films during heating or spray drying. Hydrocolloids such as guar gum, xanthan gum, gum arabic and carboxymethyl cellulose can increase continuous-phase viscosity and provide steric stabilization, but excessive addition may induce depletion or bridging flocculation and impair powder dispersion. Phosphate salts, especially dipotassium phosphate, are commonly used to buffer pH and improve resistance to protein precipitation in hot acidic coffee; nevertheless, excessive ionic strength may screen electrostatic repulsion and promote droplet flocculation. These interactions are directly related to creamer quality: controlled interfacial coverage and limited fat aggregation improve whitening and mouthfeel, whereas uncontrolled protein aggregation or droplet flocculation may cause feathering, sedimentation, oiling-off and poor reconstitution. Therefore, emulsification should be optimized by considering competitive adsorption, protein–polysaccharide interactions, salt level and final coffee/tea matrix stability rather than droplet size alone (Chung et al., 2017; Dickinson, 2003; Liu et al., 2024).

### 3.2 Emulsification

Emulsification is not only a droplet-size reduction step, but also a process of rapid interfacial formation in which plant proteins, low-molecular-weight emulsifiers and other formulation components compete or cooperate at newly generated oil–water interfaces. During homogenization or high-shear mixing, the oil phase is disrupted into small droplets, and surface-active species must adsorb quickly enough to prevent recoalescence. Plant proteins can form viscoelastic interfacial films, whereas small-molecule emulsifiers can reduce interfacial tension more rapidly and facilitate droplet breakup. However, excessive small-molecule emulsifiers may partially displace proteins from the interface, weakening protein-based protection during heating, spray drying or coffee reconstitution. Meanwhile, hydrocolloids and phosphate salts regulate continuous-phase viscosity, pH and electrostatic interactions, which can either improve stability or promote flocculation when used excessively. Therefore, emulsification conditions should be optimized by considering droplet size, interfacial composition, protein–emulsifier competition, salt level and matrix stability together. For plant-protein non-dairy creamers, the final evaluation should include not only emulsion droplet size, but also whitening capacity, feathering resistance, oiling-off and reconstitution behavior in hot coffee or tea systems (Chung et al., 2017; Liu et al., 2024; Wang et al., 2023). Table 3 summarizes the operating principles of common emulsification devices and their implications for droplet breakup and interfacial formation.

**Table 3:** Emulsification characteristics of different emulsification devices.

Emulsification devices	Emulsification mechanism	Scope of application	Average droplet size	Reference
High Shear Mixers	The rotor of the mixer induces rotational, axial, and radial velocity gradients within the system, leading to the disruption of the oil–water interface, the fragmentation of larger droplets into smaller ones, and enhanced mixing of the immiscible phases.	Preparation of coarse emulsions and dissolution of powdered components.	15–25 μm	(Koh et al., 2014)
Microjet	Fluids from two inlets are introduced at high velocity through a pumping device, causing the disruption of oil droplets and the generation of fine emulsions in a single pass.	The production of finely dispersed emulsions.	0.1–1 μm	(Taha et al., 2020)
Colloid Mill	Colloid mills generally comprise two discs: a stationary disc and a rotating disc. The crude emulsion is typically fed into the mill's central region. The rotational speed of the moving disc induces shear stresses in the narrow gap between the discs, promoting the disruption of oil droplets and subsequent emulsion formation.	Homogenization of medium and high viscosity fluids	1–2 μm	(Williams, 2001)
Membrane Emulsification	One of the immiscible liquids is forced under pressure through a microporous membrane into the continuous phase. The emulsifier is typically dissolved in the continuous phase and adsorbs at the interface, thereby stabilizing the droplets and preventing their aggregation. The droplet size can be precisely controlled by adjusting the pore size of the membrane.	This method is primarily employed at the laboratory scale to produce emulsions with high dispersed phase fractions, featuring specific sizes and structures.	0.1–2 μm	(Taha et al., 2020)

High-speed shear processing can affect the β-sheet, α-helix, and random coil structures in the secondary structure of proteins. These structural changes help improve the emulsification and solubility of proteins, enhance the stability of emulsions (Gasparre et al., 2022). Bi et al. (2018) found that shear force, during their investigation on the structural changes of soy protein isolate, could disrupt disulfide bonds, thereby exposing sulfhydryl groups associated with amino acids such as cysteine. These exposed sulfhydryl groups can undergo oxidation to form new disulfide bonds, promoting protein aggregation. As a result, the soy protein isolate adopts a more compact structure, which facilitates better dispersion of κ-carrageenan within the gel matrix. Critically, emulsification studies often vary widely in reporting of shear history (e.g., rotor–stator geometry, tip speed, and energy density), which can confound comparisons across formulations. Because high shear can both improve droplet breakup and induce protein aggregation via thiol–disulfide interchange, process optimization should consider not only initial droplet size but also interfacial integrity and viscosity evolution during storage.

### 3.3 Homogenization

Homogenization is a critical step in the preparation of oil-in-water emulsions, particularly for spray-dried coffee creamers. During emulsification, homogenization provides the high shear forces required to efficiently disrupt oil droplets, producing a fine and uniform droplet size distribution. This droplet-size reduction substantially increases interfacial area, promoting rapid adsorption of surface-active components (typically plant proteins, carbohydrates, and low-molecular-weight emulsifiers) onto newly formed interfaces. Effective interfacial coverage is essential for stabilizing droplets against coalescence and flocculation during subsequent thermal and mechanical processing (Tonon et al., 2011). Table 4 summarizes the homogenization mechanisms and applications of different homogenization devices. After high-pressure homogenization, the content of individual secondary-structure elements in the protein changes, causing changes in the arrangement of hydrogen bonds within the protein, which leads to mutual conversion between α-helices and β-sheets, making the spatial structure more stretched and increasing the irregularity of the protein.

**Table 4:** Homogenization strategies and their implications for plant-protein non-dairy creamer emulsions.

Homogenization strategy	Main mechanism	Effects on plant-protein emulsions	Advantages	Limitations / key control points	Relevance to plant-protein non-dairy creamers	References
Rotor–stator high-shear homogenization	A high-speed rotor and stator generate strong shear and turbulence, disrupting the oil phase into coarse droplets and dispersing powdered ingredients.	Promotes coarse emulsification, improves dispersion of plant proteins and wall materials, and increases contact between oil droplets and surface-active components. High shear may also change protein secondary structure or promote thiol–disulfide-related aggregation under excessive treatment.	Simple, low-cost and useful for hydration, premixing and pre-emulsion preparation.	Usually produces larger droplets than high-pressure homogenization. Rotor–stator geometry, speed, treatment time and energy input should be reported because excessive shear may induce protein aggregation.	Suitable as a pre-emulsification step before high-pressure homogenization or spray drying. It helps produce a more uniform feed emulsion, but final stability still depends on interfacial coverage and subsequent homogenization.	(Gasparre et al., 2022; Malterre et al., 2024)
High-pressure homogenization	A coarse emulsion is forced through a narrow valve under high pressure, where shear, turbulence, cavitation and impact forces break oil droplets.	Reduces droplet size, increases oil–water interfacial area, promotes adsorption of plant proteins and emulsifiers, and may alter protein secondary, tertiary and quaternary structures.	Effective for producing fine O/W emulsions with relatively narrow droplet-size distributions and improved physical stability.	Excessive pressure, repeated passes or uncontrolled temperature rise may promote protein denaturation, aggregation, weakened interfacial films or lipid oxidation. Pressure, pass number, energy density and outlet temperature should be reported.	Highly relevant to creamer feed-emulsion preparation because droplet size and interfacial coverage affect encapsulation efficiency, surface oil, oxidative stability and reconstitution behavior after spray drying.	(Guevara-Zambrano et al., 2024; Taha et al., 2020)
Microfluidization / high-pressure microjet treatment	The protein dispersion or emulsion passes through microchannels at high velocity; collision, shear and cavitation disrupt droplets or protein aggregates.	Can decrease por droplet size, improve protein dispersion, expose interfacially active groups and increase surface charge, thereby favoring aqueous dispersion and interfacial adsorption.	Useful for producing finely dispersed emulsions or nanoemulsions and for improving the functionality of poorly soluble plant proteins.	Strong mechanical input may cause over-processing, protein aggregation, temperature rise or loss of interfacial film integrity if conditions are not controlled.	Useful when small droplet size and uniform distribution are required before spray drying; also suitable as a pretreatment to improve plant-protein dispersion before emulsification.	(Gong et al., 2019; Zhao, Li, et al., 2024)
Ultrasonic homogenization	Acoustic cavitation generates local shear, microstreaming and shock waves that disrupt droplets and protein aggregates.	Can reduce droplet size, disrupt protein aggregates, expose buried hydrophobic groups and modify surface charge, thereby improving dispersion and interfacial adsorption.	Convenient for laboratory-scale screening of plant-protein emulsifying behavior and combined modification treatments.	Scale-up is limited. Excessive sonication may cause re-aggregation, oxidation, overheating or weakened interfacial film strength.	Useful for preliminary evaluation or pretreatment of plant proteins, but its industrial relevance for creamer production should be validated using scalable homogenization systems.	(Taha et al., 2020)

Disclosure statement

No potential conflict of interest was reported by the authors.

High-pressure homogenization alters protein aggregates by affecting the primary stabilizing force of hydrophobic interactions that maintain the tertiary and quaternary structures of proteins. The tertiary and quaternary structures of proteins are formed based on the secondary structure, stabilized by chemical bonds such as hydrophobic interactions, hydrogen bonds, van der Waals forces, and electrostatic interactions (Guevara-Zambrano et al., 2024).

High-pressure homogenization causes significant changes in the structure of certain plant proteins. Hu et al. (2023) reported that applying heat treatment before homogenization (20–100 MPa) increased the surface hydrophobicity of soybean protein isolate, reduced β-structure and irregular-coil contents, promoted the formation of soluble aggregates, and enhanced interfacial protein adsorption, ultimately improving emulsifying activity and stability. In contrast, heat treatment after homogenization promoted SH/SS exchange and excessive aggregation, which increased interfacial tension and could impair emulsifying performance. These findings emphasize that “more intense” homogenization is not necessarily better, especially when protein aggregation competes with interfacial coverage. This is because excessive homogenization may generate protein aggregates and alter interfacial composition, thereby destabilizing emulsions during storage. Moreover, although high-pressure homogenization is highly effective in reducing droplet size, its substantial energy input and the associated temperature rise—if not properly controlled—may accelerate protein denaturation and lipid oxidation. Therefore, studies should report homogenization pressure, energy density and number of passes to enable reproducibility and rational scale-up.

### 3.4 Sterilization

In wet-mix production of plant-protein non-dairy creamers, microbial control should be considered before spray drying, because the liquid feed contains proteins, carbohydrates and lipids that may support the growth of spoilage microorganisms during holding, transfer or delayed processing. Although spray drying reduces moisture content and water activity, it should not be regarded as a sterilization step. Therefore, heat treatment before drying should be designed not only to reduce microbial load, but also to maintain emulsion stability and prevent protein aggregation during subsequent atomization and drying (Jessica da Silva et al., 2024).

Microbial spoilage ecology is particularly relevant in wet processing systems. Spoilage bacteria such as Pseudomonas spp. can persist on stainless-steel surfaces and processing equipment by forming biofilms, which are more difficult to remove than planktonic cells (Sivri et al., 2023). Wang, Ettelaie, and Sarkar (2025) recently used ARTP mutagenesis and whole-genome analysis to identify genes involved in biofilm regulation in spoilage *Pseudomonas fluorescens* PF08, highlighting the importance of biofilm control in processing hygiene. Thus, sanitation validation for plant-protein creamer production should include verification of cleaning-in-place effectiveness, hygienic design, environmental monitoring, and control of residence time and temperature in wet-mix tanks and pipelines.

Post-drying contamination should also be considered. Low-moisture powders generally do not support microbial growth, but environmental pathogens may survive in dry products or dry processing environments and can be transferred through dust, air handling, equipment surfaces or packaging operations. For this reason, microbial safety assessment should combine feed-emulsion microbial counts, environmental monitoring, sanitation records, and finished-powder testing. At the same time, sterilization conditions should be evaluated together with emulsion and powder quality indicators, including droplet size, viscosity, ζ-potential, surface oil, oxidative stability and reconstitution behavior (Bourdichon et al., 2021).

### 3.5 Key parameters and multivariate optimization

In plant-protein non-dairy creamers, key formulation and processing parameters should be interpreted as coupled variables rather than independent factors. Oil loading, defined here as the mass percentage of oil in total feed solids unless otherwise specified, determines the interfacial area that must be covered by proteins or emulsifiers, whereas protein concentration controls both interfacial coverage and continuous-phase structuring. Total solids affect feed viscosity, atomization and pformation, while pH and ionic strength regulate protein charge, protein–polysaccharide interactions and droplet flocculation. These variables also interact with homogenization intensity: higher pressure may reduce droplet size, but this benefit depends on sufficient interfacial adsorption and suitable feed viscosity. Therefore, one-factor-at-a-time optimization may produce apparent optima that fail when oil loading, salt level, protein concentration or the final coffee/tea matrix changes (Karaca et al., 2011; Soo et al., 2021; Sriprablom et al., 2019).

Multivariate optimization methods are more suitable for plant-protein creamer systems. Karaca et al. (2011) optimized lentil- and chickpea-protein-stabilized oil-in-water emulsions using response surface methodology, with pH, protein concentration and oil loading as variables, and evaluated creaming stability, droplet size and ζ-potential. This study showed that emulsion performance was determined by combined formulation conditions rather than by a single variable. Similarly, Niroula et al. (2022) optimized pea-protein-isolate-stabilized nanoemulsions using a central composite rotatable design, in which protein concentration, oil loading and high-pressure homogenization were considered together. The model identified main, quadratic and interaction effects on droplet size and size distribution, and the optimized emulsions were further tested under electrolyte conditions. These examples indicate that future studies on plant-protein non-dairy creamers should report not only main effects, but also interaction terms such as oil loading × protein concentration, protein concentration × homogenization pressure, pH × ionic strength, and total solids × atomization conditions (Liu et al., 2018; Soo et al., 2021).

For practical formulation development, mixture design can be used to optimize the proportions of oil, plant protein, carbohydrate wall material, salts and stabilizers, while factorial, Box–Behnken or central composite designs can be used to optimize processing variables such as homogenization pressure, pass number, emulsification temperature and drying conditions. The response variables should include not only emulsion droplet size, but also ζ-potential, viscosity at process-relevant shear rates, interfacial stability, encapsulation efficiency, surface oil, oxidative indices, wettability, dispersibility, whitening capacity, feathering resistance and reconstitution behavior in hot coffee or tea. A desirability-function approach may be useful because the formulation producing the smallest droplets does not necessarily provide the lowest surface oil, best powder flowability or highest sensory acceptance. Thus, optimization of plant-protein creamers should be based on integrated emulsion-to-powder performance rather than a single quality index (Gui-jiang et al., 2019).

## 4 Processing and application of plant protein non-dairy creamer powder

**Fig. 5:**
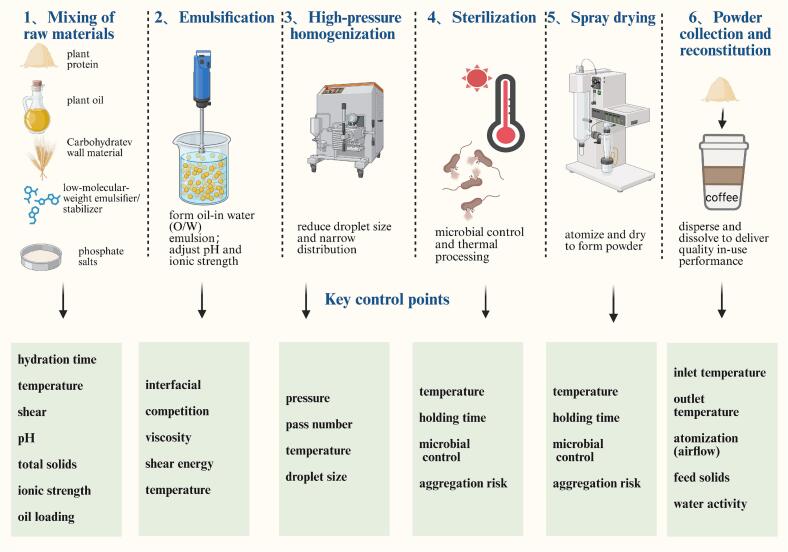
Emulsion-to-powder workflow for plant-protein non-dairy creamer production and key quality-control points.

The manufacture of spray-dried non-dairy creamers typically follows a wet-mix–homogenize–spray-dry workflow (Fig. 5). First, suitable plant proteins and other ingredients (oil phase, carbohydrates, salts/buffers, and functional additives such as emulsifiers and stabilizers) are dispersed and blended to form a pre-emulsion. This feed is then homogenized—often using a high-pressure unit that also functions as a pump—to generate a fine, stable oil-in-water emulsion with sufficient resistance to phase separation prior to drying. The homogenized emulsion is subsequently spray dried, where the key operations include atomization, rapid contact of droplets with heated air, evaporation-driven pformation, and powder separation/collection. Post-drying handling (e.g., cooling and screening, and in some processes agglomeration via a fluid-bed stage) is commonly used to improve powder stability, dispersibility, and flowability; finally, packaging under appropriate conditions helps preserve quality during storage. Importantly, these unit operations are strongly coupled: inadequate emulsion stability during wet mixing or suboptimal homogenization can propagate into variable pstructure and poorer reconstitution performance after drying, whereas careful control of drying and moisture endpoints reduces lumping and supports consistent powder functionality.

### 4.1 Spray drying of emulsion and collection of non-dairy creamer powder

Inlet and outlet air temperatures should be reported as process windows rather than isolated setpoints. For oil-containing spray-dried powders, inlet temperatures are commonly within 150–220 °C, while outlet temperatures are often controlled around 50–85 °C, depending on feed solids, oil loading, wall composition and dryer configuration (Mohammed et al., 2020). For example, virgin coconut oil powder systems relevant to creamer applications have been spray-dried at 150 ± 3 °C/70 ± 3 °C, 160 °C/50–60 °C, or 180 ± 5 °C/85 ± 5 °C. However, the optimal range should be justified by powder moisture, water activity, surface oil, oxidation stability and reconstitution performance rather than by drying temperature alone (Prasanna et al., 2024).

Drying endpoints are equally important. Spray-dried powders are generally expected to have low residual moisture and water activity; moisture contents below approximately 5% and water activity below 0.30 are commonly considered favorable for storage stability, while recent plant-based high-oil powders reported water activity values of 0.13–0.16 and moisture contents of about 4.5–4.8% (Tontul & Topuz, 2017). In addition, the glass transition temperature should be considered because water plasticization lowers Tg and promotes stickiness, caking and poor flowability during storage. Therefore, spray-drying optimization should aim to keep the product in a glassy, non-sticky state under expected storage conditions (Adhikari et al., 2025; Roos, 2002).

Surface oil should be treated as a key diagnostic indicator of spray-drying quality. High surface oil suggests insufficient interfacial coverage, droplet clustering or wall-matrix failure during dehydration, and it can reduce flowability, wettability, oxidative stability and shelf life. Because there is no universal surface-oil limit for all creamer systems, future studies should define product-specific acceptance criteria and report surface oil together with encapsulation efficiency, droplet size before and after reconstitution, and storage oxidation indices (Adhikari et al., 2025).

### 4.2 Quality analysis of non-dairy creamer

Quality assessment of spray-dried plant-protein creamers should be used as a closed-loop tool rather than a final checkpoint. The measured attributes should confirm whether the powder meets end-use specifications and, critically, provide feedback for selecting and tailoring raw materials (Section 2) and for optimizing emulsion preparation and drying conditions (Section 3 and Section 4.1). This feedback role is particularly important for plant proteins because incomplete hydration, protein aggregation, and weak or heterogeneous interfacial films can be amplified during atomization and drying, and these deficiencies often become evident only after reconstitution (Zhang et al., 2023). To translate quality data into actionable formulation and process decisions, key quality attributes can be interpreted at three levels: (i) powder state, (ii) encapsulation and storage stability, and (iii) reconstituted emulsion performance. These three levels are inherently interconnected (Gharsallaoui et al., 2007). Quality analysis of plant-protein non-dairy creamers should combine powder-state, encapsulation-stability and application-level tests. At the powder level, moisture content, water activity, bulk/tapped density, particle-size distribution, morphology, flowability, wettability and dispersibility should be routinely reported. Moisture and water activity indicate storage safety and caking risk, while pstructure and surface oil largely determine wetting and lump formation during reconstitution. Controlled agglomeration or fluidized-bed post-treatment may improve wettability, dispersibility and instant properties, but it should be evaluated together with surface oil and oxidative stability rather than treated only as a physical improvement (Adhikari et al., 2025; Hedayatnia et al., 2016).

Encapsulation and storage stability should be evaluated using both physical and chemical indicators. Encapsulation efficiency alone may overestimate product quality if surface oil remains high. Therefore, surface oil, peroxide value, p-anisidine value, TBARS, conjugated dienes or volatile oxidation markers such as hexanal should be monitored during storage. Peroxide value and conjugated dienes mainly reflect primary lipid oxidation, whereas p-anisidine value, TBARS and volatile aldehydes provide information on secondary oxidation products. These indices are especially relevant for plant-protein creamers containing unsaturated oils, because interfacial defects and exposed surface oil can accelerate rancidity (Abeyrathne et al., 2021; Drozlowska et al., 2021).

Finally, reconstitution tests should be performed in application-relevant matrices. A powder that disperses well in water may still show feathering, sedimentation, oiling-off or weak whitening in hot coffee or tea because of acidity, salts, polyphenols and heat. Therefore, future studies should report reconstitution temperature, powder dosage, beverage type, visual stability, whitening capacity, droplet-size change after reconstitution and sensory acceptability. This would make quality analysis more directly relevant to industrial creamer performance.

### 4.3 Application of plant protein non-dairy creamer

Plant-protein non-dairy creamers have been investigated as potential alternatives to conventional sodium-caseinate-based creamers in beverage and food systems. Compared with conventional formulations, their potential value lies not simply in replacing an animal-derived ingredient, but in developing an emulsion–powder system that can meet multiple application requirements, including whitening, physical stability, oxidative protection, rapid reconstitution and acceptable sensory quality (Mohammed et al., 2024). However, their practical application still depends on validated stability, sensory quality and processing performance. Proper formulation may improve mouthfeel, flavor masking and nutritional positioning, but these effects should be confirmed by sensory evaluation and application tests in realistic food matrices (Liu et al., 2023). In bread, cakes, and cookies, it has the potential to partially or completely replace milk powder and butter, using the emulsifying properties of modified plant proteins (such as pea and oat proteins) to improve product softness and reduce cholesterol content (Pourabdollah & Razavi, 2025). Several studies have explored the use of plant proteins and plant-based lipid systems in creamer-like powders or high-oil emulsion powders, but the evidence base remains limited and should be interpreted carefully. Microencapsulated virgin coconut oil-based creamer powders have been reported to improve oil handling and oxidative stability during storage, indicating the potential of spray-dried plant-lipid systems for creamer applications (Mohammed et al., 2024). More recently, plant-based high-oil powders prepared with mung bean protein isolate and maltodextrin showed that smaller emulsion droplets before drying could reduce surface oil and improve powder functionality, including dispersibility and reconstitutability (Adhikari et al., 2025). Zhiqiang et al. (2024) prepared non-dairy creamer by replacing casein with peony protein at different ratios, investigated the effects of different addition ratios on various indicators of non-dairy creamer, and evaluated the quality of non-dairy creamer made with peony protein. The results showed that the maximum replacement ratio of peony protein for casein in the preparation of non-dairy creamers was 40%. At this replacement ratio, the preparation requirements for non-dairy creamers could be met, and there was potential for application in the food industry.

These studies suggest that future plant-protein non-dairy creamers should be evaluated not only by nutritional composition or protein replacement ratio, but also by application-relevant indicators such as whitening capacity, feathering resistance, oiling-off, wettability, dispersibility, oxidative stability and performance in hot coffee or tea matrices.

## 5 Summary and outlook

Plant-protein non-dairy creamers should be regarded as multicomponent emulsion–powder systems rather than simple substitutes for sodium caseinate. Their final performance is governed by the coupled effects of protein structure, interfacial organization, formulation composition and processing history. Plant proteins can provide nutritional and sustainability-related advantages, but their direct use in creamers remains limited by poor solubility, slow interfacial adsorption, weak or overly rigid interfacial films, heat- or salt-induced aggregation, and undesirable flavor notes. Physical, enzymatic and chemical modifications can partly overcome these limitations by improving molecular flexibility, amphiphilicity, hydration and protein–oil affinity. However, excessive processing may induce aggregation, weaken interfacial film cohesion, increase bitterness or browning, and reduce digestibility or sensory quality. Therefore, the value of a modification strategy should not be judged only by solubility or emulsifying activity, but by its ability to support the whole emulsion-to-powder process.

Current evidence also shows several limitations. Many studies are still performed in simplified protein solutions or model emulsions, while fewer studies validate modified plant proteins in complete creamer formulations. Comparisons across studies remain difficult because protein source, degree of modification, oil loading, wall composition, total solids, homogenization intensity and drying conditions are often different. In addition, some studies focus mainly on droplet size or encapsulation efficiency, whereas application-relevant indicators such as surface oil, oxidative stability, wettability, dispersibility, whitening capacity, feathering resistance and reconstitution in hot coffee or tea are less consistently reported. Recent work on plant-based high-oil powders and fully plant-based spray-dried powders provides useful evidence, but further studies are still needed to connect powder structure with creamer-specific performance in real beverage systems.

Future research should focus on standardized comparison frameworks, multivariate formulation optimization and application-level validation. Protein source, degree of modification, oil loading, wall-material ratio, pH, ionic strength, homogenization intensity and drying conditions should be reported together with common quality endpoints. Response-surface methodology, mixture design or other design-of-experiments approaches should be used more widely because formulation and processing variables are strongly coupled. Mechanistic studies should further connect molecular conformation, adsorption kinetics, interfacial rheology, pformation and powder reconstitution. Finally, sensory quality, shelf-life stability, microbial control and performance in real coffee or tea systems should receive greater attention. Addressing these issues will support the rational design of stable, acceptable and industrially feasible plant-protein non-dairy creamers.

## CRediT authorship contribution statement

**Xuteng Wang:** Writing – review & editing, Writing – original draft, Visualization, Validation, Software, Resources, Project administration, Methodology, Investigation, Formal analysis, Data curation, Conceptualization. **Bingyu Chen:** Conceptualization. **Chao Yan:** Writing – original draft, Supervision, Conceptualization. **Huiwen Guan:** Conceptualization. **Mengying Liu:** Conceptualization. **Ning Li:** Resources, Conceptualization. **Hongzhi Liu:** Validation, Supervision, Funding acquisition.

## Fundings

This work was supported by 10.13039/501100001809National Natural Science Foundation of China (32472269), Beijing High-level Talent Project (19008024075).

## Declaration of competing interest

The authors declare that they have no known competing financial interests or personal relationships that could have appeared to influence the work reported in this paper.

## Data Availability

No data was used for the research described in the article.
